# Assessment of body adiposity in preterm children at the beginning of school age

**DOI:** 10.1038/s41598-019-42715-8

**Published:** 2019-04-17

**Authors:** Lidia Perenc, Katarzyna Zajkiewicz, Justyna Drzał-Grabiec, Joanna Majewska, Barbara Cyran-Grzebyk, Katarzyna Walicka-Cupryś

**Affiliations:** 1 Medical Faculty, Institute of Physiotherapy, University of Rzeszow, Rejtana 16c, Rzeszów, 35-959 Poland; 2 Centre for Innovative Research in Medical and Natural Sciences, Medical Faculty of University of Rzeszow, Warzywna 1a, Rzeszów, 35-310 Poland

**Keywords:** Medical research, Health sciences

## Abstract

In Poland, like in other developed countries, 6.3% of babies are born prematurely. Preterm babies suffer from numerous health issues. The aim of the study was to assess body adiposity in preterm children at the beginning of school age. The study population consisted of 61 children aged 5 to 8 years who had been born preterm. We performed standard anthropometric measurements according to internationally recognized methodology. The following parameters were used: Body Weight (BW), Body Heigh (BH), Waist Circumference (WC), Body Mass Index (BMI), Waist to Height Ratio (WtHR), Triceps Skinfold Thickness (TST), Subscapular Skinfold Thickness (SST), Umbilical Skinfold Thickness (UST), as well as total sum of the above parameters, or the Global Adiposity (GA). The anthropometric measurements were taken according to international anthropometric methodology. All anthropometric parameters for body adiposity were significantly lower in the study population than in the reference system. We found a statistically significant relationship between: the number of fetuses and: UST (p = 0.007) and z-score UST (p = 0.030); combined number of unfavorable perinatal events: and UST (p = 0.013) and z-score UST (p = 0.007), GA (p = 0.038) and z-score GA (p = 0.040). Preterm children who are about to start school have significantly lower values of anthropometric features that characterize their body adiposity. In preterm children at early school age number of fetuses diversifies UST; and combined number of unfavorable perinatal events diversifies UST and GA. It is recommended that more studies are conducted on positively oriented modification of body adiposity in these children, as well as its long term monitoring.

## Introduction

Infants born between 22 and 37 weeks gestational age are called preterm or premature^1^. Currently, preterm birth rate in Poland is 6.3%. This index is similar to other developed countries (5–10%)^2^. In Poland, preterm infants are given coordinated multidisciplinary care for the first three years of their life. The system of coordinated care includes, i.e. monitoring of physical development. The period of three years seems to be too short and it should be extended until the child is about to enter health school readiness phase^3^. In Poland, school readiness is assessed during one year compulsory pre-school kindergarten programme (the grade “0”) or in the first semester of the first class grade of a primary school, at the threshold of school age^4^. In Poland, anthropometric measurements of body weight and body height are used in balanced examinations in pediatrics^4,5^. One of the positive measurements of physical development is the level and the type of body adiposity. The anthropometric assessment of body adiposity measures thickness of skinfolds usually in these three points: over the triceps brachii muscle, in the umbilicus area, and at the angulus inferior of the scapula, as well as the global adiposity^6,7^. Body mass index values, related to the reference group, provide the criteria for describing the state of nourishment and its possible disorders – malnutrition and obesity^8,9^. This is due to the strong correlation between general adiposity and body mass index^10^. Anthropometric parameters, such as waist circumference and waist to height ratio, can be used to determine the distribution of adipose tissue^9,11^. However, densitometry is a more specific method; furthermore, it enables the assessment of body mass composition in children, with the inclusion of adipose tissue. The literature proves that preterm children are at risk of developing obesity in adulthood^10^. The group of newborns born prematurely is not uniform in terms of body mass at birth and gestational age. Taking into account the relationship between body mass at birth and gestational age the following can be distinguished: small for gestational age, normal for gestational age, and large for gestational age^12,13^. Children small for gestational age are known to have disrupted signal generation in response to leptin in the arcuate nucleus of the hypothalamus and reduced number of neurons of the satiety center, which results in increase in the amount of food intake. Adipose tissue in this group of children is programmed for increased proliferation and storage of fats. It is accompanied by changes in various organs associated with prematurity, including kidneys (disturbed nefrogenesis), lungs (limited development of alveoli) and blood vessels^14^. Our observations have shown that preterm children have usually poor adiposity development at the beginning of school age. Balanced examination of school readiness of children born prematurely, enriched with the measurements of thickness of three skinfolds and waist circumference, could explain the role of selected perinatal factors (unfavorable perinatal events) in differentiation of the values of some anthropometric parameters describing body adiposity^15^.

### Aim of the study

The aim of the study was to assess body adiposity in preterm children at the beginning of school age. The main goal of this study was to determine whether such factors as being born from the first or subsequent pregnancy, one- or multi-fetal pregnancy, term and way of delivery, Apgar score at fifth minute of life and birth weight, combined number of unfavorable perinatal events discriminate value of anthropometric parameters characterizing body adiposity (skinfold thickness, global adiposity, body mass index, waist circumference, waist to height ratio) in preterm children assessed before they started school.

## Material and Method

Out of 200 prematurely born children hospitalized in the Clinical Regional Hospital No. 2 in Rzeszow invited to the study, 62 children responded positively and 61 completed the full study. Finally the study population consisted of 61 children, aged 5–8 years, who had been born preterm. The study was conducted before the children started school. The significant diversity in the age of the study population resulted from the fact that some of the children were qualified to start school a year earlier, while the others obtained permission to start school a year late (6.38 years, Me = 6 years, s = 0.73). The study population consisted of 29 boys (48%) and 32 girls (52%). We conducted a perinatal questionnaire and learned that the study population were born from pregnancies of various order, e.g. first, second etc. (Table 1A), from either single or multiple pregnancy (Table 1B), of premature delivery (Table 1C), through caesarean section or vaginal delivery (Table 1D). They were born in different health condition (Table 1E) and had different body mass (Table 1F). The birth weight of the examined children was evaluated in relation to the gestational age^12,13^. The Fenton preterm growth chart was used as the reference system^13^. By plotting weight vs. gestational age, each infant is classified at birth as: Small for Gestational Age (SGA), Normal for Gestational Age (NGA) and Large for Gestational Age (LGA) (Table 1G). They had a different number of unfavorable perinatal events either (Table 1H). This indicator has already been applied^14^. Such a detailed collection of data was possible on the basis of an interview with parents and an insight into the Hospital Discharge Summary Reports they brought. Unfortunately, the attempt to collect reliable data on nutrition in infancy failed due to the inability to verify data based on medical records.

**Table 1:** Characteristics of the examined group.

A. Sequence of pregnancies			N	%
Child from I^st^ pregnancy			31	51.0
Child from II^nd^ pregnancy			15	25.0
Child from III^rd^ pregnancy			5	8.0
Child from IV^th^ pregnancy			5	8.0
Child from V^th^ pregnancy			2	3.0
Child from VI^th^ pregnancy			3	5.0
**B. Number of fetuses**			**N**	**%**
Unifetal pregnancy			39	64.0
Twin pregnancy			13	21.0
Triple pregnancy			9	15.0
**C. Time of delivery (in weeks)**			**N**	**%**
24			2	3.0
25			0	0.0
26			4	7.0
27			6	10.0
28			8	13.0
29			1	2.0
30			10	16.0
31			5	8.0
32			23	38.0
33			0	0.0
34			1	2.0
35			1	2.0
**D. Way of delivery**			**N**	**%**
Natural			10	16.0
Caesarean section			51	84.0
**E. Assessment in the Apgar scale at the fifth minute of life**			**N**	**%**
0–3			9	15.0
4–7			39	64.0
8–10			13	21.0
**F. Body mass at birth**			**N**	**%**
<750 g			3	5.0
750 g- 1000 g			10	16.0
1000 g – 1500 g			21	34.0
1500 g − 2500 g			26	43.0
2500 g- 3500 g			1	2.0
**G. Body mass at birth in relation to gestational age**			**N**	**%**
SGA - body mass at birth relation to gestational age <10 percentile			6	10.0
NGA - body mass at birth relation to gestational age 10≥, ≤90 percentile			47	77.0
LGA - body mass at birth relation to gestational age >90 percentile			8	13.0
**H. Combined number of unfavorable perinatal events (one event scores 1 point):**				
respiratory failure, respiratory distress syndrome: bronchopulmunary displasia, congenital pneumonia, pneumonia, pneumothorax, respiratotheraphy, passive oxygen therapy, hyperbilirubinaemia, anemia, thrombocytopenia, leukopenia, bleeding from respiratory, gastrointestinal, tract/cardiac tamponade, Rhesus incompability in main groups, blood or blood devirative transfiusion, exchange transfusion, hypoxic ischaemic encephalopathy, periventricular leucomalacia, intraventicular haemorrhage of I-IV degree, epilepsy, convulsions different than in epilepsy, apnea, retinopathy of prematurity, patent ductus arteriosus, TORCH infections, intrauterine infections, sepsis, purulent menangitis, encephalitis, bacterial infection of digestive system, urinary tract infection, necrotizing enterocolitis, gastro-oesophageal reflux, hipoglicemy, hipocalcemy, osteopenia of prematurity, intravenous administration of drugs, parenteral feeding, enteral feeding, procedure in general anesthesia.				
$\bar{x}$	Me	Min	Max	*s*
11.93	12.00	0	26	5.75

The approval to conduct the study was obtained from the Bioethics Commission at the Medical Faculty of the University of Rzeszow (first resolution 7/12/2012, last resolution 6/2/2017). Informed consent was obtained from parents of all participated children. The parents were present during anthropometric examinations of their children which were carried out in accordance with relevant guidelines and regulations. The study was conducted in the years 2015–2016 at the Institute of Physiotherapy of the University of Rzeszow and at the Centre for Innovative Research in Medical and Natural Sciences, Medical Faculty of University of Rzeszow.

The assessment of body adiposity based on anthropometric parameters consisted in taking measurements, calculating indices, comparing the obtained data with the reference system^5,6^ and interpreting the obtained results. The anthropometric measurements were taken according to international anthropometric methodology. We measured the following parameters: Body Weight (BW), Body Height (BH), Waist Circumference (WC), Triceps Skinfold Thickness (TST), Subscapular Skinfold Thickness (SST), Umbilical Skinfold Thickness (UST) and obtained Body Mass Index (BMI), Waist to Height Ratio (WtHR), as well as total sum of the these three skinfolds - the Global Adiposity (GA). We used medical scales (kg), anthropometer (cm), anthropometric tape (cm), the body fat caliper - Harpender spring caliper (mm). Measurement of the thickness of skinfolds was made on the right side of the body, three times, and then the arithmetic mean was calculated. The measurements were performed by an experienced pediatrician conducting research in the field of anthropometry^5,6^. We analyzed the obtained material according to the age groups and gender, along with general norms. The reference system were also the parameters presented by Perenc *et al*. 2016, in accordance with the methodology of this study^5,6^. We calculated the z-score parameter for each child. Also we categorized nourishment state in reference to the z-score BMI values interpretation of the indices^8^. The value of WtHR index >0.5 was considered as a criterion of abdominal obesity (content of visceral fat in children)^9^. The reference system did not include WC, WtHR^5,6^, but the indicated value of WtHR is a diagnostic criterion, regardless of gender and age, used in children^9^.

### Methods of statistical analysis

We conducted the statistical analysis of the obtained material in the Statictica 10.0 software package by the StatSoft company. We used the W Shapiro-Wilk test to verify if the distribution of the studied variables was normal, and the Student t-test to assess differences in the mean value of the numerical feature in two populations for independent variables, or, alternatively, the non-parametric Mann-Whithey U-test. Also, we used the one-way Anova test to assess differences in mean value of numeric feature in more than two populations, or, alternatively, the non-parametric Kruskal-Wallis Anova. In order to determine correlations of two variables that did not meet the criterion of normal distribution, we used the Spearman’s rank correlation coefficient. We used Pearson’s chi-squared test to analyze variables that were qualitative data. Statistical significance was p < 0.05.

## Results

All collected anthropometric parameters (BW, BH, BMI, TST, SST, UST, GA) were statistically significantly lower in the study population than in the growth reference values (p < 0.05) (Table 2A).

**Table 2:** Characteristics of collected anthropometric parameters related to body adiposity in the examined group (part I).

A. Comparison of anthropometric parameters of preterm children and reference system											
Variables	Preterm children					Reference system					U Mann-Whitney test (Z, p)
	$\bar{x}$	Me	Min	Max	*s*	$\bar{x}$	Me	Min	Max	*S*	
BW (kg)	21.01	20.30	13.00	40.70	4.52	24.12	24.10	18.20	32.20	3.20	Z = −4.69 p < 0,001
BH (cm)	116.59	116.00	103.00	134.50	6.47	119.51	118.50	107.90	129.90	6.26	Z = −2.21 p = 0,027
BMI (kg/m^2^)	15.36	15.26	10.46	27.80	2.43	16.67	16.90	15.50	19.10	0.59	Z = −5.38 p < 0.001
WC (cm)	53.32	53	43.00	80.00	5.58	—	—	—	—	—	—
WtHR	0.46	0.46	0.38	0.66	0.04	—	—	—	—	—	—
TST (mm)	10.72	10.00	1.00	26.00	4.37	13.40	13.40	11.10	20.40	1.92	Z = −5.09 p < 0.001
SST (mm)	7.74	7.00	4.00	23.00	3.37	8.50	8.20	6.80	12.70	1.26	Z = −3.64 p < 0.001
UST (mm)	6.74	6.00	1.00	24.00	4.21	10.49	9.80	7.70	18.40	1.91	Z = −6.22 p < 0.001
GA	25.20	23.00	10.00	73.00	10.78	32.39	31.40	26.10	51.50	4.96	Z = −6.22 p < 0.001
**B. Z-score indices of anthropometric characteristics**											
**Variables**				$\bar{x}$		**Me**		***S***		**Min**	**Max**
z-score BMI				−0.65		−0.68		1.37		−4.03	6.35
z-score TST				−0.59		−0.79		1.14		−3.64	2.80
z-score SST				−0.17		−0.29		0.93		−1.37	4.90
z-score UST				−0.68		−0.75		0.68		−2.02	2.21
z-score GA				−0.57		−0.67		0.86		−1.86	3.46
**C. State of nutrition based on the BMI z-score in the examined group**								**D. Criterion of abdominal obesity based on the value of WtHR in the examined group**			
**State of nutrition**		**Definition**			**n (%)**						
Malnutrition		z-score BMI < −1.64			7 (11.5%)			**Criterion of abdominal obesity**		**Definition**	**n (%)**
Underweight		−1.64 ≥ z -score BMI < −1			14 (23.0%)						
Correct state of nutrition		−1 ≥ z -score BMI ≤ 1			38 (62.3%)						
Overweight		1 > z -score BMI ≤ 1.64			1 (1.6%)			Unfulfilled		WtHR ≤ 0.5	57 (92%)
Obesity		z-score BMI > 1.64			1 (1.6%)			Fulfilled		WtHR > 0.5	5 (8%)
**E. Comparison of collected anthropometric parameters according to gender**											
**Variables**		**Females**			**Males**			**U Mann-Whitney test (Z, p)**			
		$\bar{x}$	**Me**	***S***	$\bar{x}$	**Me**	***S***				
BMI (kg/m^2^)		15.23	15.25	2.20	15.50	15.27	2.70	Z = 0.13 p = 0.890			
WC (cm)		52.39	52.25	5.08	54.33	53.00	6.00	Z = −1.04 p = 0.297			
WtHR		0.45	0.46	0.03	0.46	0.46	0.04	Z = −0,98 p = 0,326			
TST (mm)		11.50	12.00	4.465	9,86	9.00	4.18	Z = 1.82 p = 0.068			
SST (mm)		8.06	8.00	2.687	7.38	6.00	4.01	Z = 1.93 p = 0.054			
UST (mm)		7.03	6.00	4.076	6.41	6.00	4.40	Z = 0.63 p = 0.527			
GA		26.59	26.00	9.561	23.66	22.00	11.97	Z = 2.05 p = 0.039			
z-score BMI		−0,66	−0,58	1,29	−0,64	−0,79	1,49	Z = 1,22 p = 0,222			
z-score TST		−0.34	−0.38	1.20	−0.87	−1.07	1.01	Z = 2.69 p = 0.007			
z-score SST		−0.19	−0.08	0.68	−0.15	−0.52	1.16	Z = 1.07 p = 0.281			
z-score UST		−0.78	−0.92	0.68	−0.57	−0.73	0.68	Z = −1.42 p = 0.154			
z-score GA		−0.54	−0.54	0.79	−0.60	−0.76	0.95	Z = 1.19 p = 0.233			
**F. Comparison of collected anthropometric parameters according to sequence of pregnancy**											
**Variables**	**I** ^**st**^ **pregnancy**			**II** ^**nd**^ **pregnancy**			**III** ^**rd**^ **pregnancy & next**			**Anova Kruskal-Wallis test (H, p)**	
	$\bar{x}$	**Me**	***s***	$\bar{x}$	**Me**	***s***	$\bar{x}$	**Me**	***S***		
BMI (kg/m^2^)	15.13	15.19	1.85	16.07	15.23	3.80	15.11	15.35	1.68	H = 0.07 p = 0.694	
WC (cm)	53.04	52.50	3.73	54.97	53.00	8.28	52.23	53.00	5.54	H = 0.44 p = 0.803	
WtHR	0.46	0.46	0.03	0.47	0.44	0.07	0.45	0.46	0.04	H = 0.59 p = 0.742	
TST (mm)	11.19	11.00	4.08	11.80	10.00	4.83	8.67	8.00	4.10	H = 5.70 p = 0.057	
SST (mm)	8.10	7.00	2.94	8.33	7.00	4.59	6.40	6.00	2.56	H = 5.32 p = 0.069	
UST (mm)	6.90	6.00	3.59	7.67	6.00	5.54	5.47	4.00	3.85	H = 4.05 p = 0.131	
GA	26.19	24.00	9.4	27.80	23.00	14.07	20.53	18.00	9.61	H = 5.96 p = 0.050	
z-score BMI	−0.78	−0.74	1.19	−0.18	−0.57	2.02	−0.85	−0.72	0.85	H = 0.66 p = 0.718	
z-score TST	−0.45	−0.61	1.14	−0.34	−0.70	1.24	−1.14	−1.07	0.88	H = 4.94 p = 0.084	
z-score SST	−0.09	−0.08	0.77	0.03	−0.29	1.41	−0.54	−0.63	0.50	H = 5.27 p = 0.071	
z-score UST	−0.63	−0.73	0.61	−0.52	−0.74	0.91	−0.96	−1.00	0.52	H = 3.24 p = 0.197	
z-score GA	−0.46	−0.61	0.74	−0.35	−0.67	1.19	−1.01	−1.12	0.54	H = 4.56 p = 0.102	

The values that were below zero for $\bar{x}$ and Me - the parameters that characterize the z-score - revealed that the study population in general had lower values for BMI, TST, SST, UST, GA than the reference population (Table 2B).

We categorized nourishment state in reference to the z-score BMI values. 34.5% of the study population had body weight deficiencies (11.5% were malnourished, and 23% were underweight) and 3.2% had body weight excess (1.6% overweight, and 1.6% obesity) (Table 2C). Criterion of abdominal obesity (content of visceral fat in children) was fulfilled at 8% of respondents: one boy with obesity, one girl with overweight, two boys with correct state of nutrition, one girl with correct state of nutrition (Table 2D).

We found a statistically significant relationship between gender and GA (p = 0.039). The relationships between gender and TST (p = 0.068) and between gender and SST (p = 0.054) were close to the statistical significance. The GA value and both skinfold values were higher in females. We found a statistically significant relationship between z-score TST and the gender of the subjects (p = 0.007). Girls had values closer to 0 in the values of the growth reference charts. The remaining parameters (BMI, WC, WtHR, z-score BMI, z-score SST, z-score UST, z-score GA) were not significantly different for the two groups (p ≥ 0.05) (Table 2E).

We observed no statistically significant relationships between anthropometric parameters (BMI, WC, WtHR, UST, z-score BMI, z-score UST, z-score GA) and the number of pregnancies (p ≥ 0.05), yet we noted lower values for third and subsequent pregnancies, close to statistical significance: TST (p = 0.057), SST (p = 0.069) and GA (p = 0.050). The z-score TST relationship (p = 0.084) and z-score STT relationship (p = 0.071) were close to the significance threshold. The values for the first and second delivery were closer to the values from the growth reference charts than those for the third and subsequent deliveries (Table 2F).

There was a statistically significant relationship between UST and number of foetuses (p = 0.007) - the UST parameter decreased with increasing number of foetuses. Post-hoc test (multiple comparisons test) revealed statistically significant differences between the value of this parameter for single and triplet pregnancies (p = 0.005). It did not reveal, however, significant differences between single and twin pregnancies (p = 1.000) or between twin and triplet pregnancies (p = 0.051); though in the latter case the relationship was close to statistical significance. We found that the relationship between GA and the number of foetuses was close to statistical significance threshold (p = 0.056). The most noticeable difference for this parameter was between single pregnancies or twin pregnancies and triplet pregnancies. The relationship between z-score UST and the number of foetuses was statistically significant (p = 0.030). The more foetuses were in a pregnancy, the more the values were different from those for the growth reference system. Post-hoc test (multiple comparisons test) revealed statistically significant differences between the value of this parameter for single and triplet pregnancies (p = 0.027), yet it did not reveal differences between the values for single and twin pregnancies (p = 1.000) and twin and triplet pregnancies (p = 0.104). We found no statistically significant relationships between anthropometric features, such as: BMI, WC, WtHR, TST, SST, z-score BMI, z-score TST, z-score SST, z-score GA and the number of foetuses (p ≥ 0.05) (Table 3A).

**Table 3:** Characteristics of collected anthropometric parameters related to body adiposity in the examined group (part II).

A. Comparison of collected parameters according to number of fetuses										
Variables	Unifetal pregnancy			Twin pregnancy			Triple pregnancy			Anova Kruskal-Wallis test (H, p)
	$\bar{x}$	Me	*s*	$\bar{x}$	Me	*s*	$\bar{x}$	Me	*S*	
BMI (kg/m^2^)	15.67	15.27	2.62	14.73	15.26	2.32	14.89	15.03	1.54	H = 0.78 p = 0.674
WC (cm)	54.24	53.00	6.22	51.97	52.50	3.77	51.28	52.00	4.12	H = 1.87 p = 0.392
WtHR	0.46	0.46	0.05	0.46	0.47	0.03	0.45	0.46	0.04	H = 0.30 p = 0.859
TST (mm)	10.87	10.00	4.58	11.54	12.00	4.29	8.89	8.00	3.33	H = 3.04 p = 0.218
SST (mm)	8.21	7.00	3.57	7.54	7.00	3.43	6.00	6.00	1.58	H = 4.40 p = 0.110
UST (mm)	7.51	6.00	4.65	6.54	6.00	3.18	3.67	4.00	1.12	H = 9.80 p = 0.007
GA	26.59	23.00	11.70	25.62	24.00	9.52	18.56	18.00	5.20	H = 5.73 p = 0.056
z-score BMI	−0,46	−0,68	1,39	−1,07	−0,60	1,50	−0,88	−0,86	1,05	H = 0.76 p = 0.680
z-score TST	−0.67	−0.79	1.08	−0.15	0.03	1.28	−0.88	−1.35	1.15	H = 3.55 p = 0.168
z-score SST	−0.11	−0.26	0.96	−0.12	−0.29	1.07	−0.51	−0.63	0.50	H = 2.13 p = 0.343
z-score UST	−0.59	−0.73	0.70	−0.61	−0.73	0.68	−1.21	−1.18	0.40	H = 6.96 p = 0.030
z-score GA	−0.54	−0.67	0.87	−0.35	−0.54	0.95	−1.00	−1.12	0.55	H = 3.60 p = 0.165
**B. Dependence between collected anthropometric parameters and time of delivery**										
Spearman’s rank correlation coefficient	**Variable pairs**								**R**	**P**
	BMI vs week of delivery								−0.07	0.570
	WC vs week of delivery								−0.02	0.626
	WtHR vs week of delivery								0.04	0.766
	TST vs week of delivery								0.12	0.328
	SST vs week of delivery								−0.03	0.812
	UST vs week of delivery								0.09	0.478
	GA vs week of delivery								0.72	0.468
	z-score BMI vs week of delivery								−0.01	0.921
	z-score TST vs week of delivery								0.16	0.202
	z-score SST vs week of delivery								−0.00	0.962
	z-score UST vs week of delivery								0.14	0.268
	z-score GA vs week of delivery								0.14	0.278
**C. Comparison of collected anthropometric parameters according to way of delivery**										
**Variable**			**Natural**			**Caesarean section**			**U Mann-Whitney test (U, p)**	
			$\bar{x}$	Me	*s*	$\bar{x}$	Me	*S*		
BMI (kg/m^2^)			15.68	15.25	1.70	15.29	15.26	2.56	U = 236.0 p = 0.722	
WC (cm)			53.57	53.00	4.04	53.26	53.00	5.86	U = 235.0 p = 0.708	
WtHR			0.46	0.45	0.03	0.46	0.46	0.04	U = 237.0 p = 0.737	
TST (mm)			10.80	10.50	2.82	10.71	10.00	4.64	U = 237.0 p = 0.737	
SST (mm)			8.50	8.00	3.10	7.59	7.00	3.43	U = 195.0 p = 0.250	
UST (mm)			7.80	6.00	4.13	6.53	6.00	4.23	U = 204.0 p = 0.330	
GA			27.10	24.50	8.92	24.82	23.00	11.15	U = 210.5 p = 0.391	
z-score BMI			−0.54	−0.66	0.63	−0.67	−0.68	1.48	U = 230.0 p = 0.638	
z-score TST			−0.72	−0.70	0.50	−0.57	−0.84	1.23	U = 253.5 p = 0.977	
z-score SST			−0.22	−0.27	0.60	−0.16	−0.29	0.99	U = 250.5 p = 0.931	
z-score UST			−0.60	−0.73	0.52	−0.70	−0.82	0.71	U = 226.5 p = 0.584	
z-score GA			−0.60	−0.65	0.54	−0.56	−0.67	0.91	U = 239.0 p = 0.766	
**D. Comparison of collected anthropometric parameters according to the Apgar scale at the fifth minute of life**										
**Variable**	**0–3 pts**.			**4–7 pts**.			**8–10 pts**.			**Anova Kruskal-Wallis test (H, p)**
	$\bar{x}$	**Me**	***s***	$\bar{x}$	**Me**	***s***	$\bar{x}$	**Me**	***s***	
BMI (kg/m^2^)	15.26	15.75	2.12	15.59	15.27	2.50	14.73	14.60	2.50	H = 1.70 p = 0.425
WC (cm)	52.72	52.00	3.71	53.73	53.00	5.80	52.50	54.00	6.20	H = 0.53 p = 0.766
WtHR	0.46	0.47	0.02	0.46	0.46	0.04	0.45	0.44	0.05	H = 1.72 p = 0.422
TST (mm)	10.85	9.00	4.74	9.10	10.00	3.77	11.93	10.00	4.38	H = 3.44 p = 0.178
SST (mm)	8.15	7.00	3.29	7.05	7.00	2.42	8.07	7.00	4.02	H = 0.55 p = 0.759
UST (mm)	7.00	6.00	4.49	5.95	5.00	2.42	7.22	6.00	5.12	H = 0.11 p = 0.944
GA	26.00	21.00	11.78	22.10	23.00	6.96	27.22	24.00	12.44	H = 1.51 p = 0.469
z-score BMI	−0.77	−0.55	1.33	−0.51	−0.64	1.37	−1.00	−0.84	1.46	H = 1.16 p = 0.559
z-score TST	0.04	−0.06	0.98	−0.68	−0.84	1.09	−0.76	−1.03	1.31	H = 4.29 p = 0.117
z-score SST	−0.20	−0.06	0.42	−0.05	−0.29	1.09	−0.51	−0.52	0.55	H = 2.23 p = 0.327
z-score UST	−0.67	−0.73	0.59	−0.66	−0.76	0.74	−0.76	−0.92	0.59	H = 0.34 p = 0.842
z-score GA	−0.35	−0.28	0.49	−0.55	−0.67	0.94	−0.77	−0.91	0.81	H = 4.59 p = 0.100

It points out that a relationship was statistically significant for both the number of fetuses and UST (p = 0.007) as well also the number of fetuses and z-score UST (p = 0.030) (Table 3A).

There were no statistically significant relationships between the gestational week of delivery and collected anthropometric parameters related to body adiposity: BMI, WC, WtHR, TST, SST, UST, GA, z-score BMI, z-score TST, z-score SST, z-score UST, z-score GA (p ≥ 0.05) (Table 3B).

We did not find statistically significant relationships between collected anthropometric parameters related to body adiposity: BMI, WC, WtHR, TST, SST, UST, GA, z-score BMI, z-score TST, z-score SST, z-score UST, z-score GA and the kind of delivery (p ≥ 0.05) (Table 3C).

We did not find statistically significant relationships between anthropometric parameters: BMI, WC, WtHR, TST, SST, UST, GA, z-score BMI, z-score TST, z-score SST, z-score UST, z-score GA and a baby’s Apgar score at their 5^th^ minute after birth (p > 0.05) (Table 3D).

Also, there were no statistically significant relationships (p ≥ 0.05) between birth weight and collected anthropometric parameters related to body adiposity: BMI, WC, WtHR, TST, SST, UST, GA, z-score BMI, z-score TST, z-score SST, z-score UST, z-score GA (Table 4A), and between body mass at birth in relation to gestational age: SGA, NGA, LGA and collected anthropometric parameters related to body adiposity: BMI, WC, WtHR, TST, SST, UST, GA, z-score BMI, z-score TST, z-score SST, z-score UST, z-score GA (Table 4B).

**Table 4:** Characteristics of collected anthropometric parameters related to body adiposity in the examined group (part III).

A. Comparison collected anthropometric parameters according to body mass at birth										
Variables	<1500 g			1500–2500 g			2500 g<			Anova Kruskal-Wallis test (H, p)
	$\bar{x}$	Me	*s*	$\bar{x}$	Me	*s*	$\bar{x}$	Me	*s*	
BMI (kg/m^2^)	15.03	14.93	1.72	14.95	15.34	1.57	15.83	15.43	3.16	H = 0.79 p = 0.671
WC (cm)	52.60	52.00	4.53	52.17	52.50	3.90	54.56	53.50	6.91	H = 1.58 p = 0.453
WtHR	0.45	0.45	0.03	0.45	0.46	0.03	0.46	0.46	0.06	H = 0.78 p = 0.453
TST (mm)	12.89	13.00	3.79	10.54	10.00	4.47	9.77	9.00	4.28	H = 3.78 p = 0.150
SST (mm)	7.78	7.00	2.39	8.05	7.00	3.80	6.77	6.00	2.42	H = 1.26 p = 0.531
UST (mm)	7.56	5.00	4.07	6.64	6.00	4.39	6.46	5.00	3.97	H = 0.41 p = 0.813
GA	28.22	28.00	8.27	25.23	23.00	11.66	23.00	22.00	9.65	H = 3.59 p = 0.166
z-score BMI	−0.98	−0.90	0.87	−0.73	−0.67	1.01	−0.43	−0.60	1.77	H = 2.00 p = 0.366
z-score TST	−0.83	−1.03	0.68	−0.98	−0.70	1.12	−0.17	−0.84	1.22	H = 2.99 p = 0.224
z-score SST	−0.34	−0.29	0.52	−0.26	−0.28	0.59	−0.02	−0.52	1.25	H = 0.05 p = 0.971
z-score UST	−0.78	−0.92	0.58	−0.72	−0.82	0.44	−0.61	−0.73	0.88	H = 0.52 p = 0.769
z-score GA	−0.77	−0.76	0.60	−0.74	−0.67	0.56	−0.34	−0.67	1.09	H = 1.74 p = 0.417
**B. Comparison of collected anthropometric parameters to SGA, NGA, LGA**										
**Variables**	**SGA**			**NGA**			**LGA**			**Anova Kruskal-Wallis (H, p)**
	$\bar{x}$	**Me**	***s***	$\bar{x}$	**Me**	***s***	$\bar{x}$	**Me**	***s***	
BMI (kg/m^2^)	13.88	14.00	0.97	15.46	15.27	2.59	15.85	15.91	1.64	H = 4.28 p = 0.117
WC (cm)	49.83	50.00	6.05	53.54	53.00	5.47	54.64	53.25	5.62	H = 2.38 p = 0.302
WtHR	0.44	0.44	0.04	0.46	0.46	0.04	0.46	0.44	0.05	H = 3.47 p = 0.176
TST (mm)	8.50	8.00	3.33	10.77	10.00	4.51	12.13	10.00	3.98	H = 3.04 p = 0.218
SST (mm)	6.17	7.00	1.72	7.83	7.00	3.55	8.38	8.00	3.16	H = 1.94 p = 0.378
UST (mm)	6.50	6.50	1.87	6.55	5.00	4.48	8.00	8.50	3.89	H = 3.05 p = 0.217
GA	21.17	23.50	5.23	25.15	23.00	11.41	28.50	27.00	9.77	H = 2.15 p = 0.117
z-score BMI	−1.43	−1.07	1.58	−0.58	−0.67	1.45	−0.46	−0.45	1.04	H = 4.20 p = 0.122
z-score TST	−1.11	−1.16	1.06	−0.59	−0.70	1.12	−0.22	−0.85	1.27	H = 1.98 p = 0.370
z-score SST	−0.54	−0.42	0.57	−0.17	−0.29	0.98	0.08	0.03	0.83	H = 2.29 p = 0.317
z-score UST	−0.67	−0.79	0.33	−0.74	−0.88	0.72	−0.37	−0.42	0.66	H = 3.53 p = 0.171
z-score GA	−0.89	−0.74	0.61	−0.59	−0.70	0.88	−0.23	−0.51	0.89	H = 2.51 p = 0.285
**C. Comparison between collected anthropometric parameters and time of delivery**										
**Spearman’s rank correlation coefficient**									**R**	**P**
Variable pairs: combined number of unfavorable perinatal events vs time of delivery									−0.71	p < 0.001
**D. Comparison between collected anthropometric parameters and number of unfavorable perinatal events**										
Spearman’s rank correlation coefficient	**Variable pairs**								**R**	**P**
	BMI vs combined number of unfavorable perinatal events								−0.06	0.621
	WC vs combined number of unfavorable perinatal events								−0.07	0.589
	WtHR vs combined number of unfavorable perinatal events								−0.06	0.622
	TST vs combined number of unfavorable perinatal events								−0.15	0.242
	SST vs combined number of unfavorable perinatal events								−0.24	0.062
	UST vs combined number of unfavorable perinatal events								−0.32	0.012
	GA vs combined number of unfavorable perinatal events								−0.27	0.038
	z-score BMI vs combined number of unfavorable perinatal events								−0.05	0.688
	z-score TST vs combined number of unfavorable perinatal events								−0.10	0.447
	z-score SST vs combined number of unfavorable perinatal events								−0.19	0.149
	z-score UST vs combined number of unfavorable perinatal events								−0.34	0.007
	z-score GA vs combined number of unfavorable perinatal events								−0.26	0.040
**E. Correlation of WC, WHtR, GA and BMI in boys and girls from the examined group**										
**Boys in the examined group**						**Girls in the examined group**				
**Spearman’s rank correlation coefficient (R, p)**						**Pearson’s linear correlation (r, p)**				
**Variables**		**BMI**				**Variables**	**BMI**			
		**R**		**p**			**R**		**p**	
WC		0.57		0.001		WC	0.83		<0.001	
WtHR		0.54		0.002		WtHR	0.80		<0.001	
GA		0.56		0.001		GA	0.72		<0.001	

We found statistically significant relationships between the gestational week of delivery and combined number of unfavorable perinatal events (p < 0.001). The lower was the gestational week of delivery, the higher was combined number of unfavorable perinatal events (Table 4C).

We found a statistically significant relationship between UST and combined number of unfavorable perinatal events (p = 0.012), GA and combined number of unfavorable perinatal events (p = 0.038), z-score UST and combined number of unfavorable perinatal events (p = 0.007), z-score GA and combined number of unfavorable perinatal events (p = 0.040). There were no statistically significant relationships between combined number of unfavorable perinatal events and collected anthropometric parameters related to body adiposity: BMI, WC, WtHR, TST, SST, z-score BMI, z-score TST, z-score SST (p ≥ 0.05) (Table 4D).

Both the relationship between combined number of unfavorable perinatal events and UST (p = 0.007), as well as GA (p = 0.038) and also combined number of unfavorable perinatal events and z-score UST (p = 0.030), and z-score GA (p = 0.040) were statistically significant (Table 4D).

There was a statistically significant correlation between BMI and WC, WHtR and GA both in boys and girls (p < 0.05) (Table 4E).

## Discussion

In both female and male children in the age group from five to eight years, rather stable rate of BMI, TST, SST, UST and GA skinfold growth was observed^5,6^. It was noted that, statistically, in the examined group of preterm children the absolute values for BMI, thickness of skinfold of TST, SST, UST and GA were significantly lower than in the reference groups. Other studies present that in a group of preterm children aged two, the WC was significantly greater and the BMI was similar to the parameters shown in growth reference charts of the peers. In children aged five, WC was similar, whereas the BMI was significantly lower. A conclusion has been made that this state indicates an increase of content of visceral fat in preterm children^11^. What is more, a study on five-year-old preterm children has demonstrated that there is a similarity in GA (based on measurements of ten skinfolds) of extremely premature children (born before 26 weeks gestational age) and children born in the 26^th^ gestational week and later. No difference in visceral fat distribution has been shown by an additional measurement of WC^16^. 34.5% of the examined group had body weight deficiencies (11.5% were malnourished, 23.0% were underweight) and 3.2% had body weight excess (1.6% overweight, 1.6% obesity). Criterion of abdominal obesity (content of visceral fat in children) was fulfilled in 8.0% of respondents. A strong correlation between BMI and WC, WHtR and GA was found in examined boys and girls.

Statistically significant correlation was shown between gender and GA, and z-score TST. In females the results were closer to the values of the reference charts. The fact is, that the appearance of excessive adipose tissue in groups of children and adolescents depends both on the age and gender. For example, in Portugal it was observed that in children over nine years of age obesity increased in females and decreased in males^17^. In another study, not only anthropometric methods, but also densitometry was used to find a contradictory relationship. Preterm born adults, particularly male, presented with significantly increased fat content, distribution of which pointed to abdominal obesity. Similarly, disadvantageous location of adipose tissue was then found in their children born on time. At the same time, the study has proven the influence of preterm birth of a parent on obesity in their child^10^. It has also been revealed that higher risk of obesity generally occurs in preterm children or children with macrosomia, whose mothers had been diagnosed with obesity^18^. These examples present the influence of paragenetic and genetic factors on the process of ontogenesis^19^.

The way in which z-score TST and z-score SST were related to the number of pregnancy, was close to statistical significance. Lower values of such parameters were obtained in children from the third and subsequent deliveries. Furthermore, it has been shown that the number of pregnancies, but also the mother’s age, education, kind of her occupation and material status are the factors which influence her dietary habits^20^. As it has been found, subsequent children (born on time) appear to have tendency to be larger in size^19^. Moreover, women born from first pregnancies present greater risk of obesity than their sisters from subsequent pregnancies^21^. Higher likelihood of obesity is also characteristic of daughters from first pregnancies of obese mothers. These examples emphasise the need of educational strategies aimed at generations to curb the obesity cycle which happens repeatedly^22^.

As it has been found, body sizes of 20.0% of children from multiple pregnancies were lower than the body sizes of the children from single pregnancies^23^. Our study revealed that significantly lower UST and z-score UST appeared in preterm children from multiple pregnancies.

In children from caesarean sections, a greater risk of developing obesity in preschool age was revealed^24^. In our study, no correlation was found between gestational week of delivery or the kind of preterm delivery, and BMI, WC, WtHR, TST, SST, UST, GA, z-score BMI, z-score TST, z-score SST, z-score UST, z-score GA at the beginning of school age.

We did not find statistically significant relationships between anthropometric parameters: BMI, WC, WtHR, TST, SST, UST, GA, z-score BMI, z-score TST, z-score SST, z-score UST, z-score GA and a baby’s Apgar score at their 5^th^ minute after birth. The results of the questionnaire on the indicative study of motor and psychosocial development in terms of school readiness are differentiated by the post-natal Apgar score (in the areas of child’s functioning – high motor skills, visual-motor coordination, memory and total score) in examined group. The lower baby’s Apgar score at their 5^th^ minute after birth, the lower results of the questionnaire^15^.

Also, there were no statistically significant relationships (p ≥ 0.05) between birth weight and collected anthropometric parameters related to body adiposity: BMI, WC, WtHR, TST, SST, UST, GA, z-score BMI, z-score TST, z-score SST, z-score UST, z-score GA (Table 4A), and between body mass at birth in relation to gestational age: SGA, NGA, LGA and collected anthropometric parameters related to body adiposity: BMI, WC, WtHR, TST, SST, UST, GA, z-score BMI, z-score TST, z-score SST, z-score UST, z-score GA. The results of the questionnaire on the indicative study of motor and psychosocial development in terms of school readiness are differentiated by birth weight (in terms of hand-and-eye coordination) in this study group. The lower the birth weight, the lower results of the questionnaire^15^. The fact is, that the activity of fat tissue is reflected by adipokines concentration in blood. Preterm birth and low birth weight, in relation to gestational age, in children from both single and multiple pregnancies, are connected with lower adiponectin value^25^. As it is known, adiponectin enhances insulin sensitivity, resistin increases insulin resistance, and leptin has anorexigenic activity^26^.

There is also a connection between the energy intake and expenditure. Adipose tissue development is stimulated by excessive consumption of energy rich food^27^. There is a relationship between lowered general function and increased skinfold thickness (in particular, on the trunk), in preschool children^28^. On the contrary, it has been revealed that body adiposity is decreased by increasing physical activity in children from eight to twelve years of age^29^. The necessity of introduction of dietary supplements appears in children with low birth weight, malnutrition and delayed growth process, followed by the brain growth and neurodevelopmental deficits. Along with increased growth process, introduction of dietary supplements causes increase in visceral fat, and, in consequence, higher risk of metabolic and cardiovascular complications. The mechanism of this effect is connected to secondary hyperinsulinemia, to increased insulin resistance and down regulation of β_3_-adipocyte adrenoceptor^30^. Also, careful observation of body mass is crucial during the introduction of dietary intervention in preterm children^31^. Our study did not analyze the relationship between infant nutrition, diet and physical activity and collected anthropometric parameters. In the United Kingdom, and numerous other countries, it is recognised that educative actions should be performed in families with preschool children, to level socioeconomic differences and in order to properly maintain the nutritional status of these children^32^.

It has been found that at their 30^th^ month of age preterm children with high medical risks had significantly lower body height and head circumference and they are at risk for intraventricular hemorrage grade III or IV, periventricular leukomalacia, and bronchopulmunary dysplasia, more often than children born on time with low medical risk^33^. We found statistically significant relationships between the gestational week of delivery and combined number of unfavorable perinatal events. The lower was the gestational week of delivery, the higher was combined number of unfavorable perinatal events. In addition, a statistically significant relationship was detected between UST, z-score UST, GA, z-score GA and combined number of unfavourable perinatal events in preterm children at the beginning of school age.

The value of the study is the number of parameters we analyzed. So far, there has not been a study that would analyze all these parameters simultaneously. Also, a significant value of the study is the fact that it was conducted on preterm children as they were entering school age. Until now, most of such studies were conducted on preterm children by the age of three. The limitation of the study is relatively small study population.

## Conclusions

1. Preterm children who are about to start school have significantly lower values of anthropometric features that characterize their body adiposity.
2. In preterm children at the beginning of school age: number of fetuses diversifies UST; combined number of unfavorable perinatal events diversifies UST and GA.
3. It is recommended that more studies are conducted on positively oriented modification of body adiposity in these children, as well as its long term monitoring.
