# Author Correction: Assessment of body adiposity in preterm children at the beginning of school age

**DOI:** 10.1038/s41598-019-50174-4

**Published:** 2019-09-27

**Authors:** Lidia Perenc, Katarzyna Zajkiewicz, Justyna Drzał-Grabiec, Joanna Majewska, Barbara Cyran-Grzebyk, Katarzyna Walicka-Cupryś

**Affiliations:** 1 Medical Faculty, Institute of Physiotherapy, University of Rzeszow, Rejtana 16c, Rzeszów, 35-959 Poland; 2 Centre for Innovative Research in Medical and Natural Sciences, Medical Faculty of University of Rzeszow, Warzywna 1a, Rzeszów, 35-310 Poland

Correction to: *Scientific Reports* 10.1038/s41598-019-42715-8, published online 17 April 2019

The original version of this Article contained an error in the title of the paper, where the word “in” was omitted. This has now been corrected in the PDF and HTML versions of the Article.

